# Low Susceptibility of Rubella Virus in First-Trimester Trophoblast Cell Lines

**DOI:** 10.3390/v14061169

**Published:** 2022-05-27

**Authors:** Ngan Thi Kim Pham, Quang Duy Trinh, Kazuhide Takada, Shihoko Komine-Aizawa, Satoshi Hayakawa

**Affiliations:** Department of Pathology and Microbiology, Division of Microbiology, Nihon University School of Medicine, Tokyo 173-8610, Japan; pham.thikimngan@nihon-u.ac.jp (N.T.K.P.); takada.kazuhide@nihon-u.ac.jp (K.T.); aizawa.shihoko@nihon-u.ac.jp (S.K.-A.)

**Keywords:** rubella, trophoblast, first trimester, HTR-8/SVneo, Swan.71, susceptibility, resistance, flow cytometry, correction

## Abstract

We recently published an article about myelin oligodendrocyte glycoprotein-independent rubella infection of keratinocytes in vitro, in which first-trimester trophoblast cells were shown as rubella virus (RuV)-resistant. Given an incident rate as high as 90% of congenital rubella syndrome in the first eight weeks of pregnancy, the RuV infection of first-trimester trophoblasts is considered key to opening the gate to transplacental transmission mechanisms. Therefore, with this study, we aimed to verify the susceptibility/resistance of first-trimester trophoblast cell lines, HTR-8/SVneo and Swan.71, against RuV. Cells cultured on multi-well plates were challenged with a RuV clinical strain at a multiplicity of infection from 5 to 10 for 3 h. The infectivity was investigated by immunofluorescence (IF) assay and flow cytometry (FCM) analysis. Supernatants collected during the post-infection period were used to determine virus-progeny production. The scattered signaling of RuV infection of these cells was noted by IF assay, and the FCM analysis showed an average of 4–5% of gated cells infected with RuV. In addition, a small but significant production of virus progeny was also observed. In conclusion, by employing appropriate approaches, we determined the low infectivity of RuV in first-trimester trophoblast cell lines but not resistance as in our previous report.

## 1. Introduction

Rubella virus (RuV) is a member of the family *Matonaviridae* and the genus *Rubivirus* [1,2]. Although a clinically mild, self-limited illness with fever and a generalized erythematous maculopapular rash is often noted in children with RuV infection, the virus can cause congenital rubella syndrome (CRS), a severe complication in pregnant women, especially if the infection occurs in early pregnancy. CRS occurs after the transplacental transmission of RuV during the first eight weeks of gestation in up to 90% of cases and during the second trimester in 25–35% [3]. CRS has severe medical and public health consequences with its typical symptoms, including cataracts, congenital heart disease, sensorineural hearing impairment, hepatosplenomegaly and microcephaly, underlining the need for and importance of rubella-containing vaccines in preventing rubella and CRS [4,5,6,7,8].

Although most studies attribute fetal susceptibility to RuV-related teratogenesis in the first trimester of pregnancy to the critical periods of major organogenesis, the mechanisms of fetal RuV infection are not completely understood. Trophoblast cells have been known to be resistant to infection against various viruses, including RuV. In a recently published article about myelin oligodendrocyte glycoprotein-independent rubella infection of keratinocytes in vitro, we also reported that first-trimester trophoblast cell lines showed rubella virus (RuV) resistance [9,10].

Given an incident rate as high as 90% of CRS occurring in the first eight weeks of pregnancy, RuV infections of first-trimester trophoblasts are considered keys to opening the gate to the mechanisms of this transplacental transmission. Therefore, to provide a firm background for further studies on the mechanisms of transplacental infection by RuV, we aimed to verify the susceptibility/resistance of first-trimester trophoblast cell lines, HTR-8/SVneo and Swan.71, against RuV. By challenging cells with a RuV clinical strain at a multiplicity of infection (MOI) from 5 to 10 to ensure every single cell of the first-trimester trophoblast cell lines could theoretically come into contact with one virus particle, we clarified the low susceptibility of the cells to RuV. Therefore, the publication of this work also serves to correct our earlier understanding of the infectivity of RuV in first-trimester trophoblast cell lines.

## 2. Materials and Methods

### 2.1. Cell Culture

HTR-8/SVneo cells were originally obtained from human first-trimester placentas and immortalized via transfection with a cDNA construct encoding the simian virus 40 large T antigen [11]. Swan.71 cells (Sw.71) were derived from the telomerase-mediated transformation of a 7-week cytotrophoblast isolate described by Straszewski-Chavez [12]. These two cell lines were kindly provided by Dr. Gil Mor (Wayne State University, Detroit, MI, USA). The cells were cultured in RPMI 1640 medium (Gibco-Invitrogen, Tokyo, Japan) supplemented with 10% fetal bovine serum (FBS), 10 mM HEPES (Invitrogen), 0.1 mM nonessential amino acids (Invitrogen), 1 mM sodium pyruvate (Invitrogen) and 100 units/mL penicillin–streptomycin (complete medium). Vero cells were purchased from the Japanese Collection of Research Bioresources Cell Bank and cultured in Dulbecco’s modified Eagle medium (DMEM) (Gibco-Invitrogen, Tokyo, Japan) supplemented with 10% FBS and 100 units/mL penicillin–streptomycin. All cells were cultured in monolayers at 37 °C in a humidified 5% CO_2_ incubator. All the experiments in this study, including virus infection, were carried out in the biosafety level 2 laboratories of Nihon University School of Medicine, Tokyo, Japan.

### 2.2. Rubella Virus

The clinical RuV strain (3-B1-RK13) was transferred from Kitasato University School of Medicine (Tokyo, Japan). The viral stock solution was prepared by propagating the virus in Vero cells and concentrating the viral particles via ultracentrifugation at 52,000× *g* for 90 min in a Himac CS100GX micro-ultracentrifuge with an S50A rotor (Hitachi Koki Co., Ltd., Ibaraki, Japan). Viral titers were estimated with the 50% tissue culture infectious doses (TCID50) method or flow cytometry (FCM) analysis.

### 2.3. Viral Infection

The cells were cultured in 96-well plates (5 × 10^3^ cells/well, for FCM analysis) or 6-well plates with glass coverslips (10^5^ cells/well, for immunofluorescence (IF) assay). On day 2 post-seeding, the cells were washed with serum-free medium and then incubated with the virus at multiplicities of infection (MOI) from 5 to 10 for a total of 3 h at 35 °C, in a humidified 5% CO_2_ incubator with gentle shaking every 10–15 min during the first hour. The supernatant was removed; then, the cells were washed, and the medium was replaced with a fresh medium containing 2% FBS. The percentage of cells infected with the virus was determined from 24 to 48 h post-infection (hpi) by FCM analysis and 48 hpi by IF assay. Negative-control cells (mock-infected, infected with heat-inactivated RuV or not exposed to the primary antibody during the staining procedures) and positive controls (RuV-infected Vero and/or A549 cells [13,14,15]) were prepared in parallel for comparison. Supernatants were collected daily and replaced with fresh medium until day 5 pi to monitor viral progeny production.

The infectivity of the collected supernatants was determined by the TCID50 method or FCM analysis. For the TCID50 method, briefly, 1 day before infection, Vero cells were seeded onto 96-well plates (10^4^ cells/well). Serially, 10-fold-diluted virus stocks or supernatants (100 μL/well) were inoculated in the cells in quadruplicate. Then, 50 uL of fresh medium containing 2% FBS was added every 5 days, and cytopathic effects were monitored under microscopy. The results were collected on day 14 post-infection (pi) after fixation and staining with crystal violet. The TCID50 was calculated using the Reed–Muench method [16].

For FCM analysis, 30 μL of serial 3-fold dilutions of the supernatants was used to infect (in duplicate) freshly seeded Vero cells in a 96-well plate (4 × 10^4^ cells/well). Medium containing 2% FBS and NH4Cl was added 6 hpi to prevent a second round of infection (final concentration of NH4Cl, 20 mM). The cells were collected 24 hpi and subjected to intracellular staining of RuV capsid protein. The viral titer (in infectious units (IUs)) of a sample was calculated as the average of 3 titers measured in 3 consecutive wells with a percentage of RuV-infected cells lower than 40% and higher than 0.3%, as described previously [17,18].

### 2.4. Immunofluorescence Assay

The cells cultured on glass coverslips in six-well plates were subjected to RuV infection as described above. Separate negative controls subjected to mock treatment, heat-inactivated RuV inoculation and staining with normal mouse serum were established. The supernatant was removed 48 hpi, and the cells were fixed with cold methanol for 5 min, washed with PBS and incubated with mouse monoclonal anti-RuV capsid antibody (ab34749; Abcam, Waltham, MA, USA) for 1 h at RT. The cells were washed with PBS and incubated with an Alexa 488-conjugated goat anti-mouse IgG (H + L) secondary antibody (ab150117; Abcam) solution for 30 min at RT. The samples were counterstained with 4′,6-diamidino-2-phenylindole dihydrochloride (DAPI) (Lonza, Walkersville, MD, USA). After washing, the coverslips were mounted with VECTASHIELD Mounting Medium (Vector Labs, Burlingame, CA, USA), and fluorescence images were acquired using a fluorescence microscope (FLoid Cell Imaging Station; Life Technologies, Carlsbad, CA, USA).

### 2.5. FCM Analysis

The studied trophoblast cells were collected from 24 to 48 hpi via trypsinization or using a detachment medium (RPMI containing 2.9 mM EDTA, 2% FBS, Live/Dead Staining Solution (Live/Dead Fixable Near-IR Dead Cell Stain Kit; Thermo Fisher Scientific, Waltham, MA, USA)). For the latter, the cells were incubated in an incubator for approximately 1 h after adding the detachment medium [19]. After washing with a staining buffer (STB, cold PBS containing 5% FBS and 2 mM EDTA), the cells were fixed with paraformaldehyde and permeabilized using a BD Cytofix/Cytoperm Fixation/Permeabilization Solution Kit (BD Biosciences, San Diego, CA, USA). Intracellular staining was performed with mouse monoclonal anti-RuV capsid antibody (ab34749; Abcam) for 30 min at RT. The cells were washed and incubated with a goat anti-mouse IgG H&L (Alexa Fluor^®^ 647) secondary antibody solution (ab150115; Abcam) for 30 min at RT. They were then subjected to FCM analysis after washing and fixation. For each sample, at least 5000 gated events were collected and analyzed on a BD FACSVerse cytometer using BD FACSuite software (version 1.2; BD Biosciences). Negative-control groups without viral inoculation or incubated with heat-inactivated RuV were established.

To investigate the cell-surface expression of E-cadherin and cytokeratin 7, the cells were stained and collected using a previously described two-step protocol for preparing adherent cells [19]. Briefly, the supernatant was removed, the detachment medium (as described above) and primary antibodies were added and incubated in an incubator for approximately 1 h. After one wash with STB, cells were incubated with goat anti-rabbit IgG H&L (Alexa Fluor 488) secondary antibody (ab150081). The cells were washed, fixed with paraformaldehyde and subjected to FCM analysis or to intracellular staining for the RuV capsid protein.

### 2.6. Statistical Analysis

An analysis of variance was used to analyze the results. A *p*-value of <0.05 obtained using the Tukey–Kramer test and Statcel 4 software (OMS Publishing, Inc., Tokorozawa, Saitama, Japan) was considered significant. Data are presented as the mean ± SEM.

## 3. Results

**Figure 1:**
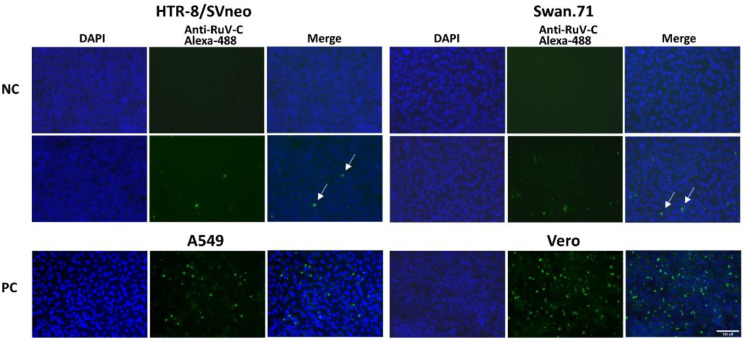
Microscopic images of the first-trimester trophoblast cells infected with RuV. The cells were fixed 48 hpi and labeled with mouse monoclonal anti-rubella viral capsid antibody, followed by Alexa 488-conjugated goat anti-mouse IgG (H + L) secondary antibody (green). Nuclei were stained with DAPI (blue). A549 and Vero cells were used as positive controls. Trophoblast cells that were mock-infected, incubated with heat-inactivated RuV, or stained with mouse serum were used as negative controls. Images are representative of 3 independent experiments. RuV-C, rubella virus capsid; NC, negative control using heat-inactivated RuV; PC, positive control. Scale bar: 100 μM.

In this study, the scattered intracellular localization of RuV capsid protein in first-trimester trophoblast cells was noted by immunofluorescence (IF) assay. Compared with the positive controls, A549 and Vero cells, the densities of the IF signals of the studied trophoblast cells were much lower. In addition, the IF signals often appeared in smaller spots, implying possible limited replication of RuV in these infected trophoblast cells (Figure 1).

**Figure 2:**
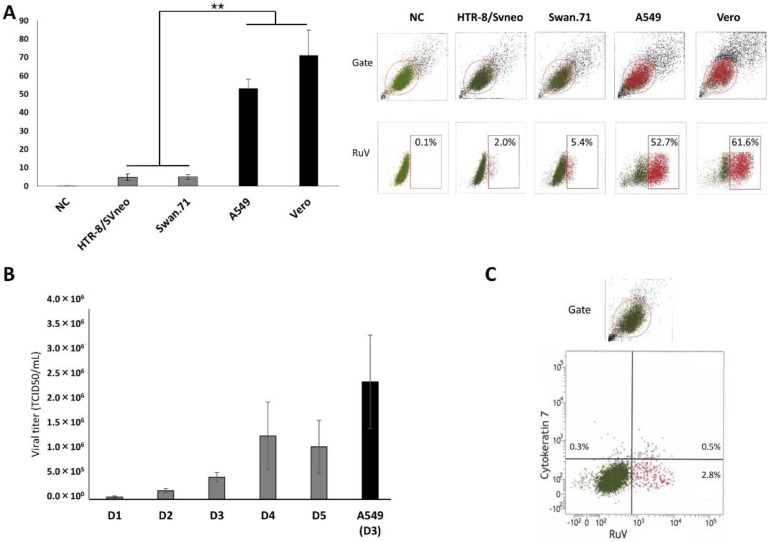
(**A**) Percentages of RuV-positive gated trophoblast cells determined by FCM analysis and their representative images. Trophoblast cells and reference cells cultured in 96-well plates were incubated with RuV at an MOI from 5 to 10. The cells were harvested from 24 to 48 hpi, stained with anti-RuV capsid protein antibody and then with Alexa-647-conjugated second antibody. The numbers displayed inside each panel correspond to the percentage of the cells positive for RuV capsid protein of the parent gated population. The results are expressed as the mean (±SEM) of at least triplicate experiments in each group, and the graph is representative of three independent experiments. ** *p* < 0.01. (**B**) Titers of viral progeny in RuV-infected Swan.71 cell supernatants collected from day 1 to day 5 pi determined by TCID50. The titers of supernatants collected from RuV-infected A549 cells on day 3 pi were used as a positive control. NC, negative control. (**C**) No exclusive association between RuV-infected HTR-8/SVneo trophoblast cells and cell-surface expression of cytokeratin 7.

The infection with RuV in the studied trophoblast cells was confirmed by FCM analysis. The percentages of RuV-infected trophoblast cells ranged from 2% to 7% (4–5% on average) of gated cells obtained from various repeated infection experiments. Much higher percentages of RuV-infected cells were observed in the positive controls, more than 50% and 60% for A549 cells and Vero cells, respectively (Figure 2A). The successful replication of RuV in trophoblast cells was observed with a 5-day monitoring of viral progeny released into supernatants, with the peak occurring on day 4 post-infection (Figure 2B). We detected almost no cell-surface expression of E-cadherin on the studied cells (less than 0.3%, similar to the percentage of cells exhibiting background staining) based on FCM analysis. The cell-surface expression of cytokeratin 7 was found in small percentages (from 1 to 3% for HTR-8/SVneo cells) and was not exclusively associated with the RuV-infected trophoblast cells (Figure 2C).

## 4. Discussion

Because trophoblasts are barriers between the mother and the fetus, they play an essential role in protecting the fetus from potential viral infections. Therefore, determining the infectivity of RuV in trophoblast cells is indispensable for further research into the mechanisms of RuV transplacental infection. In this study, trophoblasts were incubated with rubella virus at a high MOI from 5 to 10 to ensure that every trophoblast cell would theoretically come into contact with at least one virus particle. Consequently, the low infectivity of RuV in first-trimester trophoblast cell lines, HTR-8/SVneo and Swan.71, was established with the evidence of RuV infection obtained by IF assay and FCM analysis and of viral progeny production post-infection. Such findings were not observed in our previously published study. In that study, this low infection was missed because the virus stock solution used in the infection step was not concentrated by ultracentrifugation, resulting in much lower MOIs (0.1–0.2) being applied [10]. The results of this study improve the current understanding of the mechanisms of RuV infection of the fetus in the first trimester of pregnancy. Up-to-date, well-established literature shows that CRS occurs at a rate of approximately 90% during the first trimester; however, well-established evidence of RuV infection of first-trimester trophoblasts has not been reported. Taken together with the previous report of RuV infection of endothelial cells [20], although found in low infectivity rates, this in vitro finding implies that the placenta has a possibility of infection. The finding appears to be in concordance with the clinical observation that fetal RuV infection is not observed in every RuV-infected pregnant woman.

Including our study findings, the current understanding suggests that there may exist unknown factors which facilitate fetal RuV infection. Compared to the in vitro experimental conditions, the natural intrauterine environment with different levels of oxygen tension during placental formation, nutritional conditions, metabolic activities and underlying maternal diseases may favor infection. Regarding this hypothesis trend, our latest finding shows that RuV infection of these first-trimester trophoblast cells could be enhanced under low-glucose stress conditions [21].

In line with the above discussion, this study has the natural limitation of in vitro experiments using immortalized human trophoblast cells. Therefore, the low infectivity of these trophoblast cells in artificial cultures in relation to actual clinical conditions must be interpreted appropriately. Future studies employing explants or other trophoblast cells in intrauterine-mimicking culture conditions should be considered. In addition, trophoblasts function as a natural barrier, secreting interferons to protect the fetus from viral infections [22]; mechanisms of the possibly limited replication of RuV in infected trophoblast cells should be investigated.

## 5. Conclusions

In this study, by using appropriate approaches, we confirmed the low infectivity of RuV in first-trimester trophoblast cells studied in vitro. The findings provide a firm background for further studies on the mechanisms of transplacental infection of RuV. In addition, the results of this study amend our understanding of RuV infection of first-trimester trophoblasts in vitro.

## Data Availability

The raw data supporting the conclusions of this article can be made available by the authors, without undue reservation.
